# Classical Models of Hydroxide for Proton Hopping Simulations

**DOI:** 10.1021/acs.jpcb.4c05499

**Published:** 2024-12-03

**Authors:** Ankita Dutta, Themis Lazaridis

**Affiliations:** † Department of Chemistry and Biochemistry, City College of New York/CUNY, 160 Convent Avenue, New York, New York 10031, United States; ‡ Graduate Program in Biochemistry, The Graduate Center, City University of New York, 365 Fifth Avenue, New York, New York 10016, United States; § Graduate Programs in Chemistry and Physics, The Graduate Center, City University of New York, 365 Fifth Avenue, New York, New York 10016, United States

## Abstract

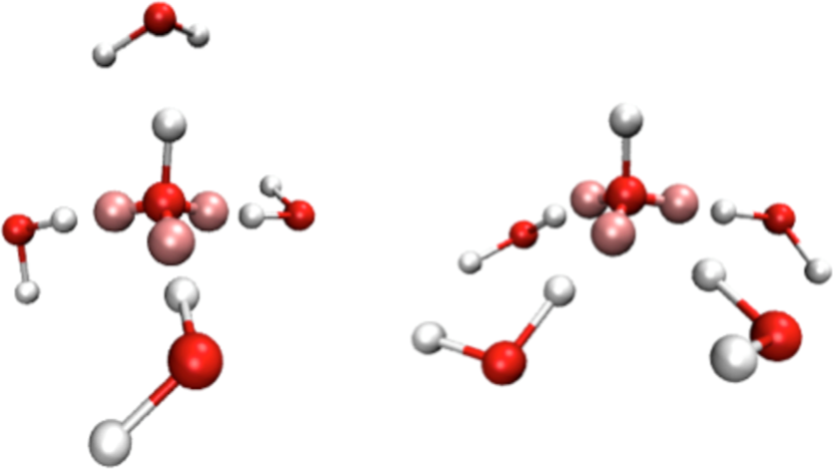

Hydronium (H_3_O^+^) and hydroxide (OH^–^) ions perform structural diffusion in water via sequential proton transfers (“Grotthuss hopping”). This phenomenon can be accounted for by interspersing stochastic proton transfer events in classical molecular dynamics simulations. The implementation of OH^–^-mediated proton hopping is particularly challenging because classical force fields are known to produce overcoordinated solvation structures around the OH^–^ ion. Here, we first explore the ability of two-particle point-charge models to reproduce both the solvation free energy and coordination number in TIP3P water. We find that this is possible only with unphysical changes in the nonbonded parameters which create problems in proton hopping simulations. We then construct a classical OH^–^ model with the charge of oxygen distributed among three auxiliary particles. This model favors a lower coordination number by accepting three hydrogen bonds and weakly donating one. The model was implemented in the MOBHY module of the CHARMM program and was fit to reproduce the experimental aqueous diffusion coefficient of OH^–^. This parameterization gave reasonable electrophoretic mobilities and the expected accelerated transport under nanoconfinement.

## 1 Introduction

H_3_O^+^ and OH^–^ ions, products of water autoionization, play critical roles in chemistry and biology. First, their relative abundance influences chemical reactivity and regulates the structure and function of biological macromolecules (for example,^1^). Moreover, free energy in living systems is initially stored in the form of a proton electrochemical gradient across the inner membrane of mitochondria and chloroplasts.^2^ The mechanisms of generation of this gradient and its subsequent usage to generate ATP^3^ or perform other useful work^4^ are fundamental problems in Bioenergetics. To understand these problems, one needs to describe proton movement through proteins and across membranes. In this process, H_3_O^+^ and OH^–^ are equally important; standard electrophysiology experiments cannot distinguish between movement of H_3_O^+^ in one direction and movement of OH^–^ in the opposite direction.^5^

It is now well-established that proton movement in aqueous environments occurs by sequential proton transfers (“Grotthuss hopping”).^6−8^ This makes it difficult to study with classical molecular dynamics (MD) simulations. Various solutions have been proposed, such as QHOP,^9^ λ-dynamics,^10^ reactive force fields,^11^ and multistate empirical valence bond theory (MS-EVB).^12−14^ A few years ago, our lab proposed a simple method (MOBHY, for MOBile HYdrogen) that allows mutual change in the protonation state for hydrogen-bonded titratable residues during a classical MD run.^15^ A similar approach was more recently presented for ionic liquids.^16^ The current implementation of MOBHY includes hydronium, His, Glu, and Asp and has been applied to the study of H^+^-mediated proton hopping in several membrane protein systems.^15,17−19^ The goal of the present work is to extend the scope of the method by developing a classical OH^–^ model suitable for proton hopping simulations.

Two distinct solvation structures of the OH^–^ ion in an aqueous medium have been identified by pioneering Car–Parrinello ab initio molecular dynamics (AIMD): a tetracoordinated (4CN) square planar structure in which the oxygen of OH^–^ accepts 4 hydrogen bonds (HB) from the neighboring waters, and a tricoordinated (3CN) structure in which the oxygen of OH^–^ accepts 3 HB and the hydrogen of OH^–^ weakly donates one.^20−22^ The planar 4CN structure is predominant in aqueous solution, whereas the tetrahedral 3CN structure is the state that fosters proton hopping due to its topological similarity to a water molecule (“presolvation concept”). According to this “dynamic hypercoordination” theory, reaching the 3CN state from the 4CN state is the rate-limiting step for hydroxide mobility. Another AIMD study with a different functional found the 3CN state to be dominant,^23^ but that functional predicts an OH^–^ diffusivity higher than that of H^+^, in contrast to experiment.^24^ More recent studies with advanced functionals confirmed the hypercoordination but not the coplanarity of the coordinating waters.^25^ A wider range of coordinations was observed in low-temperature water clusters using enhanced sampling.^26,27^

Neutron diffraction experiments analyzed with an empirical potential structure refinement protocol confirmed the 4CN structure with weak HB donation by the hydroxide, except that the four waters were found to be noncoplanar.^28^ The coordination number (CN) decreased at higher ion concentrations. Notably, a fractional charge for both OH^–^ and Na^+^ was used in the Monte Carlo simulations to fit the data in these studies. Tetracoordination of OH^–^ without HB donation was also supported by X-ray absorption spectroscopy of 4 and 6 M aqueous KOH solutions,^29^ an FTIR study of alkali metal hydroxides,^30^ X-ray diffraction,^31^ and core-hole deexcitation spectroscopy.^32^ Dielectric relaxation spectroscopy, analyzed with several assumptions, gave a hydration number of 5.5.^33^ On the other hand, vibrational spectroscopy in small gas-phase clusters supported a 3CN structure for the OH^–^ solvation shell.^34^ 2D-IR in solution was unable to distinguish between 3CN and 4CN states^35^ but provided valuable data on the dynamics of proton transfer.^36^

OH^–^ models in classical force fields typically produce an excessive CN above 6.^37−40^ Reduction of the net charge of the ion produced results more in line with experiment.^28,41^ A more recent parameterization effort with a full net charge aimed to reproduce the solvation free energy (SFE) and the activity derivatives, but the CN and the solvation structure were not reported.^42^ Various models targeting the SFE were explored by Lee and Meuwly.^43^ Polarizable models for OH^–^ in polarizable water have also been proposed,^44,45^ including a model with five auxiliary sites targeting the CN and solvation structure observed in AIMD simulations.^46^ A nonpolarizable model with the negative charge distributed to a ring of 20 massless particles around the oxygen along with other modifications in nonbonded interactions was also able to produce a lower CN^47^ and served as the basis of an EVB model.^48^ A couple of MS-EVB models were also developed by the Voth group^14,49,50^ but they are more complex and have been used much less than the same group’s excess proton models.

The goal of the present work was to identify and implement OH^–^ models appropriate for MOBHY proton hopping simulations in biomolecules.^15^ Therefore, we restricted attention to the TIP3P model for water, which is widely adopted in the parameterization of biomolecular force fields, and set as parameterization targets the SFE and the solvation structure around the ion, partially described by the CN. We first considered simple 2-particle models, allowing the variation of the net charge. We then developed a 5-particle model with the charge on the oxygen of OH^–^ divided into 3 auxiliary particles (3AP). We found that the 3AP model favors a tight 3CN tetrahedral state with 3 HB accepted and 1 HB transiently donated, which is consistent with the solvation structures of the OH^–^ ion primed for the acceptance of a proton hop. The new OH^–^ models were implemented in the MOBHY module of the CHARMM program and were tested in bulk water and narrow carbon nanotubes (CNTs).

## 2 Methods

### 2.1 Hydroxide Model Development and Testing

Classical MD simulations were performed with the CHARMM package. Bulk water systems were created by adding 1 OH^–^ ion to a water box containing 924 or 995 TIP3P water molecules (corresponding to pH ∼ 12.7). All bonds involving hydrogen atoms were constrained using SHAKE. The systems were minimized using CHARMM’s adopted basis Newton–Raphson (ABNR) and steepest descent (SD) algorithms, followed by 600 ps dynamics under constant temperature and pressure using the Hoover thermostat^51^ and the Langevin Piston barostat.^52^ The cutoff for the van der Waals interaction was set at 12.0 Å. Periodic boundary conditions (PBC) were used and long-range electrostatic interactions were calculated using PME.^53^ No counterion was included in the unit cell, as we previously found that a small net system charge has no discernible effect on proton dynamics^18^ (see also Figure S1). O_H_–O_w_ radial distribution functions (RDFs) and HB distributions were obtained using VMD.^54^

**Figure 1:**
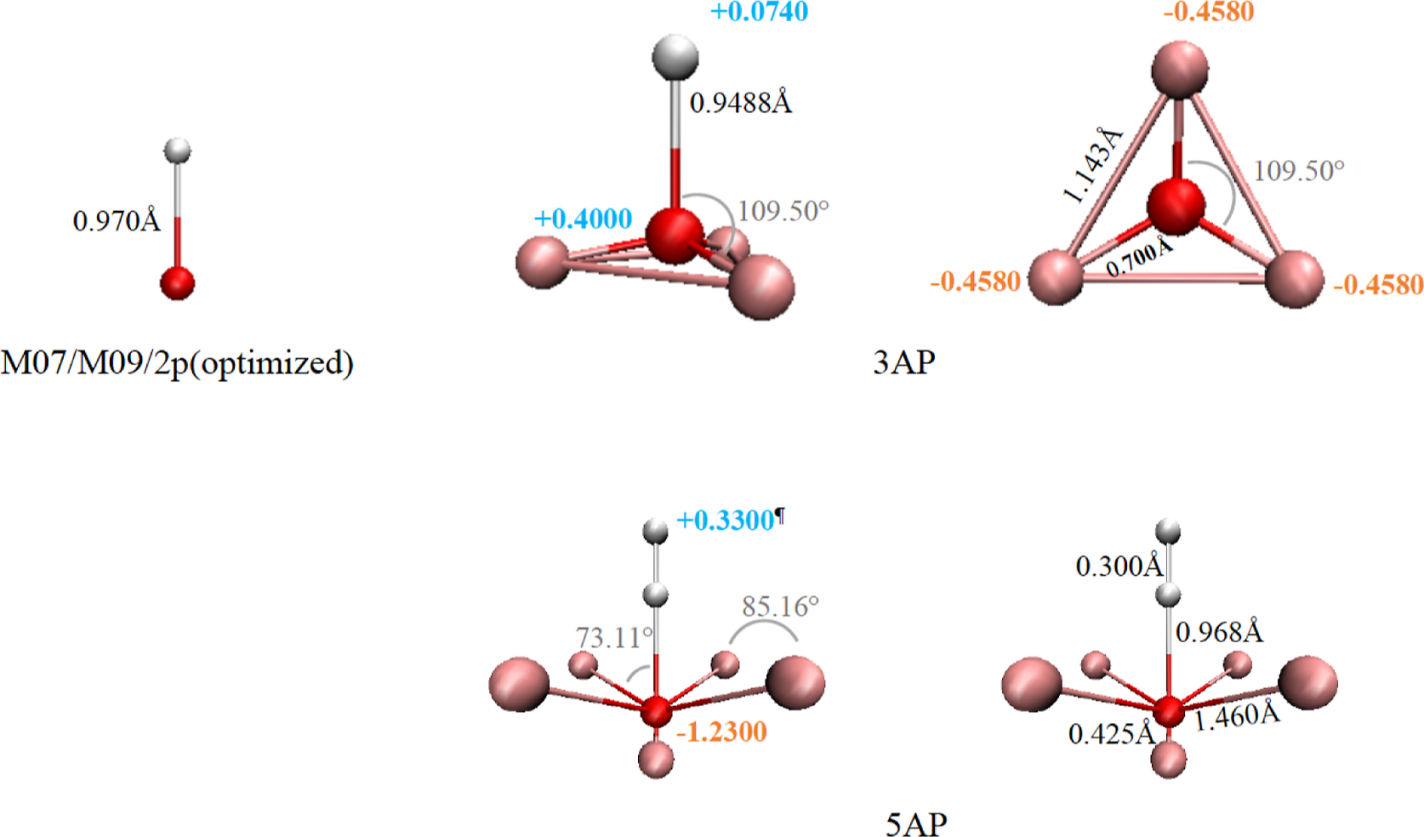
Molecular models used in this work. Oxygen and hydrogen atoms are shown as red and white spheres, respectively. APs are shown as pink spheres. Partial charges are shown in colored font, angles are shown in gray, and bond lengths are shown in black font. The symbol ^¶^ refers to the site where a hydrogen charge is placed in the 5AP model.

Two sets of OH^–^ models were considered in this study: two-particle (2p) models, with the partial charges placed on oxygen and hydrogen atoms, and AP models (Figure 1). The AP models tested can be further divided into two categories: One with oxygen nonbonded interaction terms distributed among 5 APs and one with only the oxygen charge distributed among 3 APs. The former AP model is a variant of a polarizable OH^–^ model^46^ and the latter was inspired by the charged ring model.^47^ Standard Lorentz–Berthelot combination rules were used for the OH–water nonbonded parameters.

For the 2p models, systematic optimization of the Lennard-Jones parameters was carried out starting from three sets of values (Table 1) using a SD algorithm^55,56^
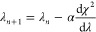
1where λ_*n*+1_ is the new value and λ_*n*_ the old value of parameter λ, α is an empirically chosen step size, and dχ^2^/dλ is the derivative of the target function with respect to the parameter of interest.

**Table 1:** Initial Sets of Force Field Parameters That Were Used for the Parameter Space Scan (*r*_min_ = 1.122462 σ)^a^

	charge		well depth (ε_o_)		*r*_min_/2	
source	O	H	O	H	O	H
original CHARMM	–1.30	0.30	0.12	0.046	1.70	0.2245
Ufimtsev et al.^47^	–1.074	0.074	0.024	0.046	1.98	0.2245
Lee and Meuwly^43^	–1.183	0.183	0.06	0.044	1.75	1.443
TIP3P	–0.834	0.417	0.1521	0.046	1.7682	0.2245

a The corresponding values of the CHARMM-modified TIP3P model are given on the last line.

We define the target function χ^2^ to be minimized as the sum of the squares of the difference between experimental (*A*_exp_) and observed (*A*_obs_) values of certain observables (*A*), so dχ^2^/dλ can be obtained as follows
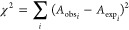
2
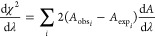
3where d*A*/dλ refers to the partial derivative of *A* with respect to a force field parameter to be optimized. The two observables of interest here were (1) the CN, i.e., the area under the first peak of *g*(*r*), and (2) the magnitude of the first peak of *g*(*r*) (referred to as the “Peak” hereafter). The d*A*/dλ term was obtained according to eqs S1–S7 (see Supporting Information).

The SFE for all models was obtained by performing alchemical free energy simulations using the PERT module in CHARMM. Bulk water systems were set up as described above. The perturbation scheme attempted for the 2p and AP models was slightly different. For the 2p model, λ = 0 was defined as a system of 924 TIP3P waters and λ = 1 as a system of 923 waters and 1 OH^–^ ion. For this setup, the self-solvation free energy of TIP3P water (−6.10 kcal/mol^57^) was added to the final SFE. The SFE of the AP model was calculated by switching all constituent atoms of the AP model into dummy atoms, thereby eliminating all nonbonded interactions of the AP OH^–^ ion. Therefore, λ = 0 was defined as a system consisting of 923 TIP3P waters and 1 AP OH^–^ ion, while λ = 1 was defined as a system of just 923 TIP3P waters and 1 noninteracting AP OH^–^ ion. The free energy perturbations were performed over 10 windows during a 110 ps dynamics run. Each window was 10 ps long, including 2 ps of equilibration. All calculations were repeated three times, and the standard deviation of the three runs was taken as the statistical uncertainty.

The SFE of single ions cannot be determined experimentally but can be estimated based on extrathermodynamic assumptions. One widely accepted value for OH^–^ is −105 kcal/mol.^58,59^ This estimate is from gas-phase cluster studies and includes a surface potential contribution of about ±12 kcal/mol, with sign opposite to the sign of the ion.^60−62^ Since the simulations here were run under PBC, the values calculated do not include this contribution and correspond to the “intrinsic” SFE, which for OH^–^ should be around −117 kcal/mol. Considering the variation in proposed values for the SFE of H^+^,^60−64^ values between −112 and −117 kcal/mol for the intrinsic SFE of OH^–^ should be considered acceptable.

### 2.2 Proton Hopping Simulations Using MOBHY

Proton hopping simulations via the OH^–^ ion were carried out using the MOBHY method^15^ in a cubic periodic box of edge length 31 Å. Proton hops were considered only between molecules that are H-bonded to each other. H bonds were identified using CHARMM’s legacy HBOND facility. Starting structures were minimized, and 1 ns long MD runs were performed at constant temperature and pressure. PME was used for long-range electrostatic interactions. Titratable HOH residues were created where the second H could be a dummy atom, in which case the residue is a hydroxide, or it can be an actual proton, in which case HOH is equivalent to TIP3P. The energy change upon a proton hop (Δ*E*_hop_) is evaluated by switching the nonbonded parameters of the two partners. The code provides a detailed decomposition of all energetic contributions to the Δ*E*_hop_ values. When the donation of a proton from TIP3P is considered, the TIP3P molecule is switched with a titratable HOH residue. Typically, about 5 HOH residues need to be present in a system for every OH^–^ ion. Proton hop acceptance occurs via a modified Metropolis criterion where Δ*Ε* is replaced by (Δ*E* – *C*), where *C* is an empirical threshold parameter chosen so as to reproduce the diffusion coefficient or hopping rate. For the AP models, the system energy was minimized after each hop acceptance using the ABNR algorithm. The number of minimization steps was chosen to approximately remove the amount of energy that the hop added to the system.

To obtain OH^–^ diffusion coefficients in bulk water, 1 ns trajectories were generated with proton hops attempted every 0.02 ps (10 MD steps). The trajectories were unwrapped to remove the effects of PBC, and a time series of the OH^–^ oxygen coordinates was obtained. ⟨*r*^2^⟩ vs time graphs were produced and the slope of the linear portion was used in the Einstein relation *D* = ⟨*r*^2^⟩/6*t* to obtain the diffusion coefficient. Electrophoretic mobility (velocity/electric field) was calculated using the same system. A potential of 1 V was applied in the *z* direction over the entire cell length of 31 Å (to compensate for the lower net charge of some models, the potential was increased to 1.1 V for models with −0.9e net charge and 1.43 for models with −0.7e net charge). Trajectories from 100 ps simulations were obtained and unwrapped as described above. The velocities of OH^–^ were calculated as the slope of the *z* coordinate vs time plot.

Pristine CNTs were used to study OH^–^ hopping in nanoconfined spaces. A 100 Å long narrow (6,6) armchair CNT was constructed as described previously.^15^ The CNT was filled with 3 or 5 titratable waters and 42 or 40 nontitratable TIP3P waters in a single file arrangement. One of the titratable waters was designated as deprotonated. Hops were attempted every 10 steps. Constraints from CHARMM’s MMFP module were used to prevent evaporation of water from the tube’s ends. Constant temperature was maintained by scaling velocities every 10 steps if the average temperature deviated by more than 5 K. The one-dimensional (1D) version of the Einstein equation *D* = ⟨*r*^2^⟩/2*t* was used to calculate the diffusion coefficient over the linear portion of the plot.

### 2.3 Analysis of the AIMD Trajectory

An ab initio simulation trajectory^65^ was graciously provided by Dr. Mark E. Tuckerman. The 50 ps microcanonical simulation used DFT-based Car–Parrinello MD with the B-LYP functional and DVR basis functions on 31 water molecules and 1 OH^–^ ion in a cubic periodic box with edge length 9.87 Å. No dispersion corrections were included in this study (more recent work that included such corrections confirmed the basic picture but produced less planar and/or more diverse solvation structures^25,27^). The trajectory was analyzed to study the solvation structures of the OH^–^ ion to compare it with classical MD trajectories.

Molecular configurations were extracted from the AIMD trajectories using a script developed in-house. To account for PBC, the script first added copies of the system around the central simulation box, producing one layer of periodic images. Hydrogens were then assigned to the closest oxygens and the oxygen with only 1 hydrogen assigned was designated as the OH^–^ ion. 50,000 coordinate frames in .*xyz* format were obtained using this method. The system was translated so that OH^–^ was always at the center of the box. The trajectories were visualized with VMD. Oxygen of hydroxide to oxygen of water (O_H_–O_W_) RDFs were obtained using VMD. The statistical uncertainty in the RDFs was estimated by splitting the trajectory into four blocks, calculating an RDF in each block, and overlaying the results (see Figure S2).

Proton hops were counted by tracing the index of OH^–^ oxygen. Rattling events (return of the donated proton to its previous oxygen partner) were distinguished from true proton hops. In this way, 15 true proton hops out of a total of 143 were identified over the 50 ps trajectory, corresponding to 3.3 ps/hop, which is in good agreement with experiment.^66^

## 3 Results

### 3.1 Development and Testing of Classical OH^–^ Models

#### 3.1.1 2-Particle, Point-Charge Models

**Figure 2:**
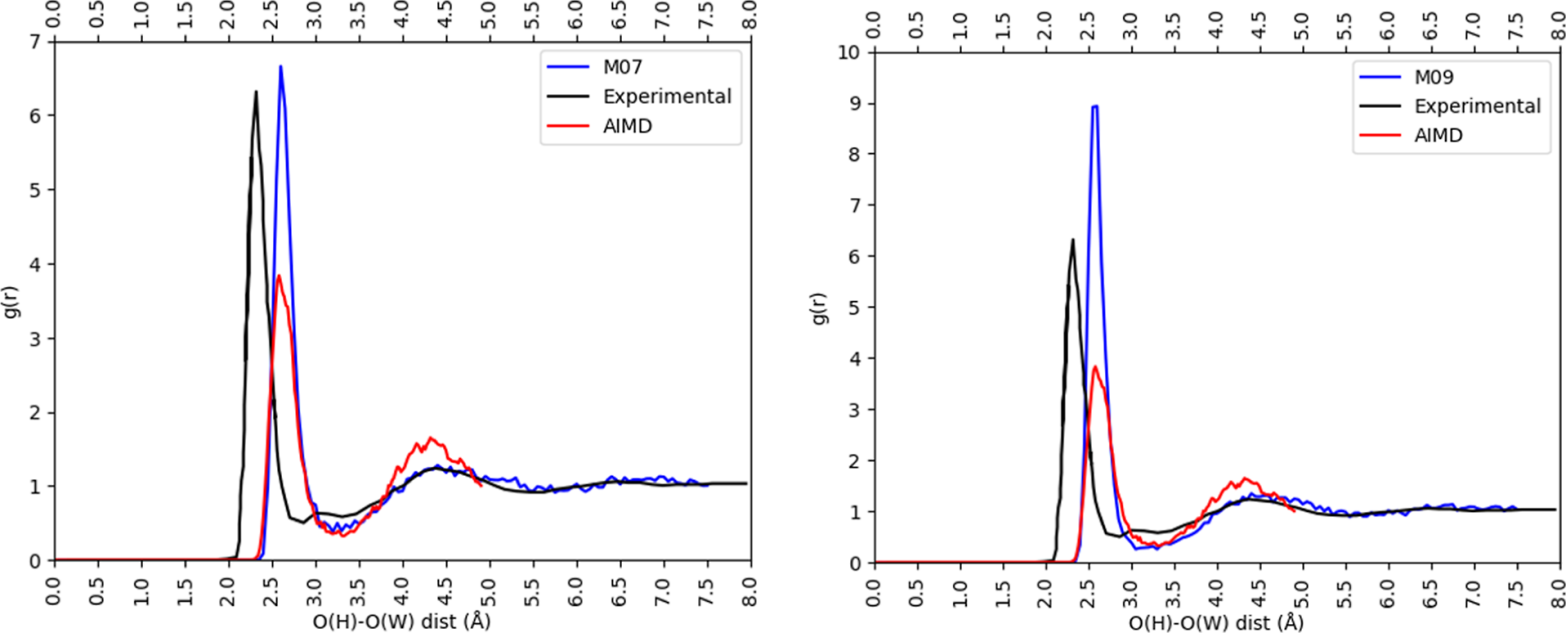
O_H_–O_W_ RDFs for the M07 (left) and M09 (right) hydroxide models compared to experiment^28^ and AIMD simulation.^65^

We started with the 2p model distributed with the CHARMM parameter set (A. MacKerell, unpublished), which has partial charges of −1.3 on O and +0.3 on H (Table 1). The SFE for this model in TIP3P water was calculated as −139.4 ± 0.3 kcal/mol, significantly more negative than the target range from −112 to −117 kcal/mol. The CN in water was 6.6, similar to other classical models.^37^ Reducing the magnitude of both partial charges reduces the SFE but leaves the CN unchanged. Reducing the total charge to −0.9, −0.8, or −0.7 gradually improves the CN but also reduces the SFE. With a charge of −1.0 for the O and +0.3 for the H, the CN was 5.6 and the solvent configuration was qualitatively similar to the square planar, 4-fold coordination reported in CPMD simulations, with an additional water donating an H bond from a direction roughly colinear with OH. However, the SFE was −70.1 ± 0.2 kcal/mol, significantly smaller than the experimental value. Further reductions in the net charge did not lead to substantial reductions in the CN. Total charges of −0.8 or −0.9 gradually increased both the CN and the SFE. Thus, we implemented two classical 2p models with reduced charges: (a) the M07 model (O: −1.0, H: +0.3) which gives better coordination (CN = 5.6) but low SFE (−70.1 ± 0.2) and (b) the M09 model (O: −1.2, H: +0.3) which gives good SFE (−113.1 ± 0.1 kcal/mol) but overcoordination (CN = 6.1). The RDFs for these two models are shown in Figure 2, together with those from the AIMD trajectory and experiment. Note that there is a substantial discrepancy between the latter, which, to our knowledge, has not been addressed in the literature. Given this discrepancy, we aimed for an RDF that is between those from AIMD and experiment.

We then explored whether a systematic variation of the LJ parameters of O and H (ε_o_, ε_H_, *R*_minO_, *R*_minH_) could result in a model that simultaneously reproduces both the CN and the SFE. First, each of the four parameters was varied independently of the others starting from the Ufimtsev parameter set (Table 1), which produced a reasonable SFE (−120.59 ± 0.09 kcal/mol) with a net charge of −1.0e. The RDFs obtained indicate that the force field modifications that produce smaller CN also produce a more rigid first hydration shell and vice versa. Increase in the magnitude of the ε_O_ and the *R*_minO_ parameters also leads to an increase in the distance of the first peak of the O_H_–O_W_ RDF (Figure S3a,b). Increasing the *R*_minH_ was the sole parameter modification that produced improved O_H_–O_W_ RDFs with a diffuse first hydration shell along with a smaller CN (Figure S3d). Note that *R*_minH_ is also larger in the Lee and Meuwly parameter set compared to the other two (Table 1).

Automatic optimization using a SD algorithm was tried next. Starting points were the Ufimtsev and the Lee and Meuwly parameter sets with an overall charge of −1.0e (Table 1). The χ^2^ values were obtained by setting the target CN to 4.5 and the peak to 4 in accordance with the AIMD results. SD optimization confirmed the ability of an increased *R*_minH_ parameter to reduce the CN (Figure S4 and Table S1). However, the optimal *R*_minH_ turned out to be larger than that of *R*_minO_, which is quite unnatural. In addition, there is a complete absence of HB donation via the hydrogen atom of OH^–^. At the local minimum of χ^2^, the parameter set obtained using the SD algorithm produced a somewhat higher Δ*G*_solv_ value of −124.0 ± 0.2 kcal/mol.

#### 3.1.2 Auxiliary Particle Models

Ufimtsev et al.^47^ proposed an OH^–^ model with a ring composed of 20 massless charged particles. This is not practical for our purposes, so we considered a smaller number of APs, each of which was given a mass of 1 amu. We started our optimization efforts with 4 APs and with an overall charge of −1.0e. Three different placements of the nonbonded parameters were tested (Table S2). Placement (1) assigns LJ parameters to the APs, whereas the other two placements to the oxygen. Placements (2) and (3) are similar, except that the latter has a small positive charge on the oxygen atom.

With all three placements, the first peak of *g*(*r*) was observed at the same distance (2.5 Å), but its magnitude was quite dissimilar (Figure S5). The smallest peak value is produced with placement (1), when all nonbonded parameters are placed on the APs. However, this placement also produces the largest CN of about 6 waters in the first hydration shell. The smallest CN is observed with placement (3), which was thus adopted for further testing.

**Figure 3:**
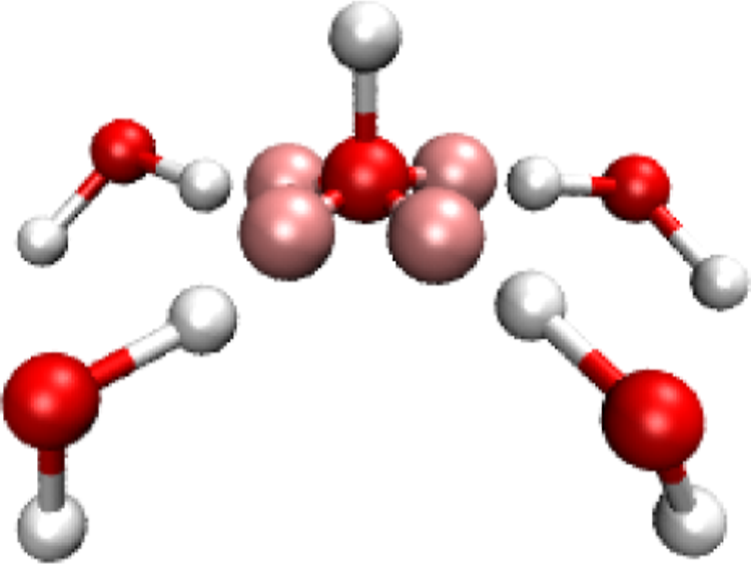
4CN solvation structure produced by the 4AP model with placement (3) and an overall charge of −0.9e. Oxygen and hydrogen atoms are shown as red and white spheres, respectively. APs are shown as pink spheres.

To further reduce the CN, we reduced the overall charge to −0.9e, which led to a modest reduction in CN. No switch between the 4CN and 3CN states was observed. In this model, each AP interacts strongly with one water molecule and thus favors 4CN solvation structures (Figure 3). While a 4CN solvation structure is a significant improvement compared to the solvation structures produced by the 2p models, proton hopping via the OH^–^ ion is known to be favored by the presolvated tetrahedral 3CN state.^22^ We therefore proceeded to create a 3AP model with placement (3) and an overall charge of −0.9e.

The 3AP model was subjected to further optimization. No changes were made to the charge of the hydrogen atom. The magnitude of positive charge on the oxygen atom has an effect on the solvation structure produced: the peak of the O_H_–O_W_ RDF plot increases and the CN decreases as the positive charge on the O (and the negative charge of the APs) is increased (Figure S6a). Modification of the oxygen to AP (O–AP) bond length was also considered for the 3AP model with a +0.4e charge on the oxygen atom. We found that an increase in O-AP bond length also produces a larger peak value and lower CN (Figure S6b). The value of 0.7 Å was chosen as the SFE obtained with this model was −115.3 ± 0.3 kcal/mol, within the expected range. The final parameter set for the 3AP model is provided in Table 2.

**Table 2:** Force Field Parameters for Hydroxide Models Tested

	M07/M09	2p-opt	3AP	5AP
*q*_o_ (e)	–1.0000/–1.2000	–1.0742	+0.4000	–1.2300
*q*_H_ (e)	+0.3000	+0.0742	+0.7400	+0.3300^a^
*q*_AP_ (e)			–0.4580	
ε_o_ (kcal/mol)	–0.1200	–0.0699	–0.0240	
ε_H_ (kcal/mol)	–0.0460	–0.0410	–0.0460	–0.0460
ε_AP_ (kcal/mol)				–0.0703
*R*_mino_ (Å)	1.7000	1.5990	1.9825	
*R*_minH_ (Å)	0.2245	2.1051	0.2245	0.3927
*R*_minAP_ (Å)				1.5708/1.3464^b^

a Indicates partial charge placed on the hydrogen charge site.

b Indicates LJ *R*_minAP_ placed on the additional AP placed 0.425 Å away from the oxygen atom (Figure 1).

**Figure 4:**
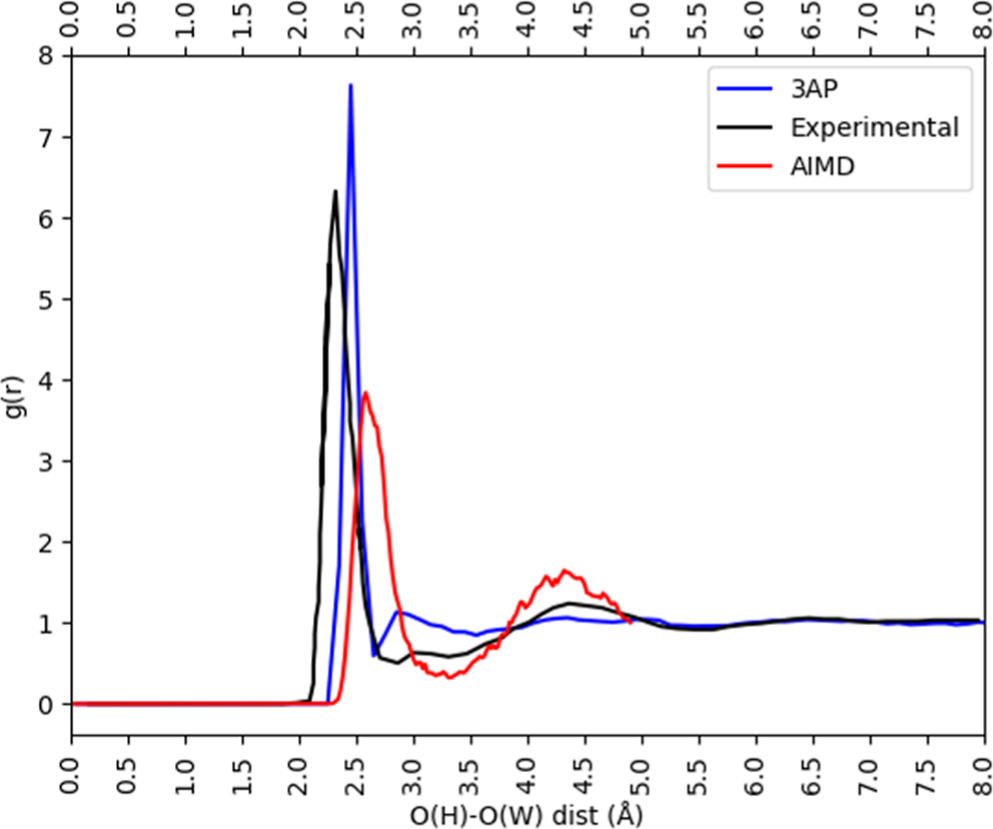
Comparison of O_H_–O_W_ RDF obtained with the 3AP model (an overall charge of −0.9e, positive charge on oxygen of +0.4e, and O–AP bond length of 0.7 Å) with O_H_–O_W_ RDF obtained using experiment and AIMD. Experimental O_H_–O_W_ RDF is based on Figure 6 of ref (28).

Figure 4 shows that the peak of the O_H_–O_W_ RDF obtained with the 3AP model is higher than those obtained from experiment and AIMD. The distance at which the first peak occurs with the 3AP model (2.45 Å) is between the values in the experimental (∼2.3 Å) and the AIMD (2.6 Å) RDF. Furthermore, a small second peak is also seen in the 3AP RDF around 2.75 Å (experimental RDF also has a smaller bump around 3 Å). Visual examination indicates that this model has predominantly 3 accepted HB in the first hydration shell at an O_H_–O_W_ distance of 2.4 Å. A fourth water molecule is also found at a larger O_H_–O_W_ distance and corresponds to either a HB donation via the hydrogen atom or a fourth HB acceptance via the oxygen atom of the OH^–^ ion.

**Figure 5:**
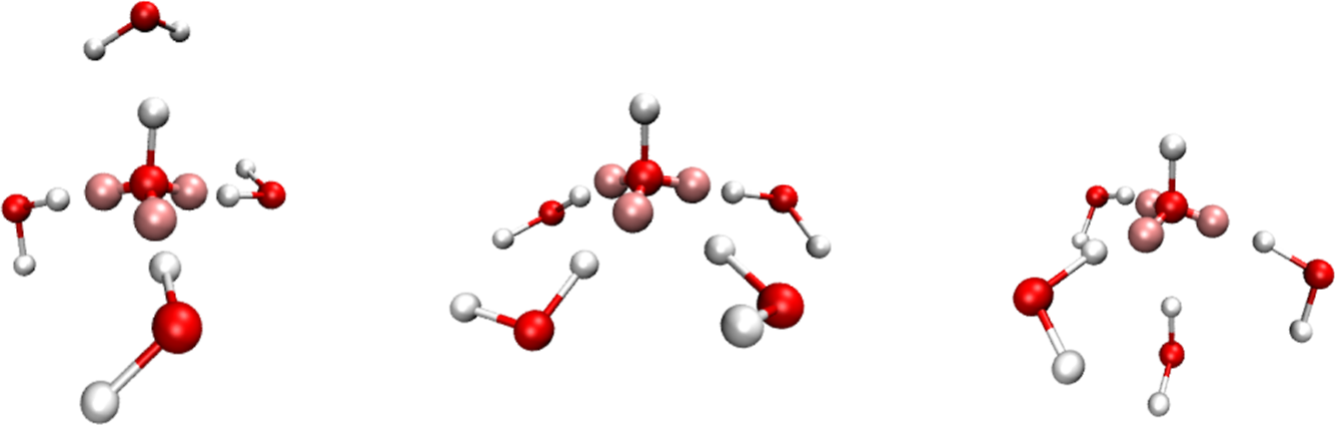
Τetrahedral (left) 3CN, planar (middle), and nonplanar (right) 4CN solvation structures observed with a 3AP model with a positive charge of +0.4e on the oxygen atom and an overall charge of −0.9e. The O–AP distance in this model is 0.70 Å. The same color scheme as Figure 3 is used.

When a fourth HB is accepted, the water molecule oriented mainly in a planar arrangement but some instances of nonplanar arrangement of HB acceptance are also seen around the oxygen of the OH^–^ ion (Figure 5). The 3AP OH^–^ with a positive charge on the oxygen atom was therefore capable of mimicking the 3CN tetrahedral structures that are expected to facilitate proton hopping via the OH^–^ ion.

A 5AP model was also constructed based on a previous polarizable model^46^ (Figure 1 and Table 2). This model was found to favor a 5CN solvation structure, accepting 4 HB and donating one (Figure S7b). The first peak of the RDF had a magnitude intermediate between experiment and AIMD but was placed at longer distances than either of these (Figure S7a). It was also found to give a very high SFE in TIP3P (−187.5 ± 0.25), so it was not pursued further.

### 3.2 Classical Proton Hopping Simulations Using the MOBHY Algorithm

Protonation state changes in MOBHY are mimicked by using special titratable molecules that allow changes in atom types and charges upon acceptance of a proton hop. The protonated form of a titratable OH^–^ ion (referred to as an HOH residue hereafter) must be identical to a TIP3P water model. This is exactly true for the 2p models but not for the 3AP model because of the mass of the APs. The solvation properties of the HOH residues were therefore tested further and the O_w_–O_w_ RDFs of a TIP3P residue and that of the HOH residue were found to be identical to each other (Figure S8).

#### 3.2.1 Diffusion Coefficient and Electrophoretic Mobility of OH^–^ in Bulk Water

The distribution of hopping energies (Δ*E*_hop_) for the M07 and M09 models, as well as the 2p-opt model of hydroxide, was obtained from MOBHY trajectories without proton hops. The minimum and median values were 4 and 34, respectively, for M07 and 24 and 66 kcal/mol, respectively, for M09. The standard, full-charge version of OH gives a distribution of hopping energies shifted to even higher values, with minimum and median values 38 and 89 kcal/mol, respectively. For M07, the value *C* = 12 gave a diffusion coefficient of 0.61 ± 0.05 Å^2^/ps and electrophoretic mobility of 18.7 ± 2.4 Å^2^/(V ps). For M09, the value *C* = 28 kcal/mol gave diffusion coefficient 0.48 ± 0.09 and electrophoretic mobility 40.2 ± 16 Å^2^/(V ps). The large variance in the last number is due to charge movement occurring in bursts, which can be different from one simulation to the next. The experimental values are 0.53 Å^2^/ps^67^ and 20.50 Å^2^/(V ps),^68^ respectively.

The average Δ*E*_hop_ for the 2p-opt OH^–^ model was 354 kcal/mol, dominated by the van der Waals energy. The reason for this exceedingly high value is the large difference in the van der Waals *R*_min_ parameter of the hydrogen atoms in the OH^–^ ion and in TIP3P water, which causes extensive clashes upon proton hopping. Thus, this model was not pursued further.

For the 3AP model in bulk water, the diffusion coefficient as a function of the C parameter is shown in Table 3. With a *C* = 35 kcal/mol, the average diffusion coefficient observed over three independent 1 ns trajectories was 0.59 Å^2^/ps with an average of 374 hops accepted (Table 3 and Figure S9). The hopping rate (∼3 ps/hop) agrees roughly with the rate of true hops observed in the AIMD trajectory. The electrophoretic mobility of the 3AP OH^–^ model calculated using the same *C* parameter was 12.2 Å^2^/ps *V* ± 3.5 (Figure S10), somewhat lower than the experimental value.

**Table 3:** OH^–^ Diffusion Coefficients for Different Threshold Values for the 3AP Model (Average of 3 Trials)^a^

*C* (kcal/mol)	average no. of accepted H^+^ hops	average diffusion coefficient (Å^2^/ps)
0	0	0.24 (±0.011)
30	59 (±8.8)	0.22 (±0.054)
32	160 (±4.5)	0.32 (±0.045)
35	374 (±48)	0.59 (±0.135)
38	2230 (±541)	0.93 (±0.657)
40	5883 (±574)	1.102 (±0.119)

a Standard deviations are provided in parentheses.

#### 3.2.2 Carbon Nanotubes

Spatial confinement can change the solvation structure of water as well as the H_3_O^+^ and OH^–^ ions.^69^ A QM/MM study of H^+^ and OH^–^ in narrow CNTs calculated diffusion coefficients of both ions much higher than in bulk water, but the absolute values were not reliable due to PBC-created defects.^70^ Interestingly, OH^–^ was found to diffuse faster than H^+^, contrary to the trend in bulk water. This study also established an average lifetime of 0.015 ps (or 0.16 ps when rattling events were excluded) for an OH^–^ embedded in a 1D water wire in the longest studied nanotubes. Another study using a dissociable water model did not use PBC and was able to calculate an absolute diffusion coefficient of OH^–^ in the longest CNTs equal to 32 Å^2^/ps,^71^ much higher than 0.53 Å^2^/ps in bulk water.^67^ In contrast, lower diffusivity than in the bulk was observed in CNTs or graphane bilayers functionalized with trimethylammonium.^72^

A large increase in the diffusion coefficient of H_3_O^+^ was observed in a (6,6) armchair CNT using the MOBHY algorithm.^15^ However, the value obtained was significantly higher than the 17 Å^2^/ps obtained by an EVB simulation.^73^ Because the hop acceptance rate in narrow CNTs is very high, the diffusion coefficient is sensitive to the protocols used. With a 40-step posthop energy minimization, the value of *C* = 23 kcal/mol that reproduces the diffusion coefficient in bulk water gives *D* = 13 ± 10 Å^2^/ps in narrow CNTs, more in line with EVB.

**Figure 6:**
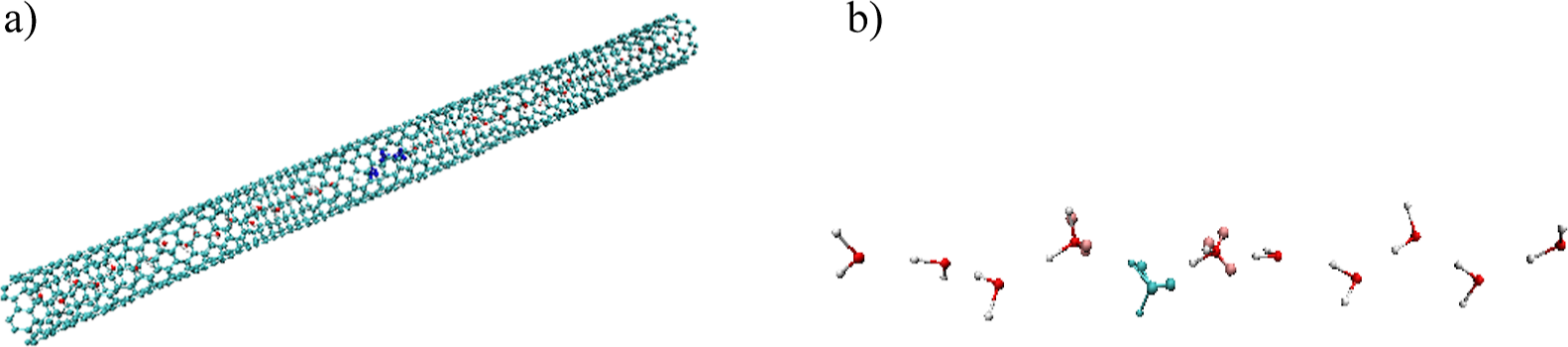
(a) (6,6) armchair CNT filled with a single file of water. (b) Representative snapshot of OH^–^ embedded in the single file of water; specialized 3AP HOH residues are depicted as described previously, and the cyan 3AP HOH molecule is deprotonated and represents the real OH^–^ ion.

We now turn to OH^–^-mediated proton hopping in a 100 Å long (6,6) armchair CNT which only allows single file water occupancy (Figure 6). The 1D diffusion coefficient obtained for the M07 and M09 models was 44 ± 0.9 and 46 ± 10 Å^2^/ps, respectively, higher than the above theoretical estimates. The 3AP OH^–^ 1D diffusion coefficient obtained in the CNT was 29 Å^2^/ps ±10, in agreement with the dissociative model results.^71^ An average rate of 0.025 ps/hop was observed in this narrow CNT. It should be noted that the results for M07 and M09 are without posthop minimization, whereas the 3AP results are with a 4-step minimization. Posthop minimization in M07 and M09 actually increases the observed diffusion coefficients because it reduces the percentage of back hops.

## 4 Discussion

In this work, we sought simple classical models for OH^–^ that would be appropriate for proton hopping simulations. The parameterization target was the SFE and the solvation structure/CN. We found that a reduction in the overall charge is effective in bringing the CN closer to neutron diffraction and DFT results. Lower CN could also be obtained artificially by increasing the *R*_minH_ parameter, but that creates serious problems in proton hopping events. More specific control of the solvation shell structure is afforded by AP models at some additional computational expense. A model with three APs mimicking the lone pairs in the Lewis structure of OH^–^ creates a solvation structure similar to the “active” structure of AIMD simulations observed during a proton hop. O_H_–O_W_ RDF with the 3AP OH^–^ model agreed reasonably well with the experiment (Figure 4).

The net charge of all proposed models is less than −1.0e. Use of these models makes the total charge of the system nonintegral, but this did not appear to create any problem in the simulations. In any case, counterions of opposite charge could be used to neutralize the system. Interestingly, it has been suggested that the lack of electronic polarization in classical nonpolarizable simulations could be accounted for by scaling the charge of ions by 1/√1.78 = 0.75 (where 1.78 corresponds to the refractive index of water).^74,75^ This was suggested to improve ion–ion interactions and the transfer free energy of ions from polar to nonpolar media, such as the membrane bilayer interior. Better agreement with experimental enthalpy of the transfer of polar ions from a high dielectric liquid to low dielectric vapor phase was obtained when roughly half of the aqueous SFE was reproduced with scaled atomic charges.^74^ The recently proposed Madrid-2019 force field also advocates for the scaling of the charge of monovalent ions by 0.85 (or 0.75 for solutions with a higher concentration of ions).^76,77^ Thus, there may be theoretical justification for the better performance of scaled charge ions, but the effects of scaling on the interaction with biomolecular groups need to be considered carefully.

Our models are parameterized in bulk water, and one may justifiably wonder how transferable they are to the various environments encountered in biomolecular simulations. As a limiting case, we considered narrow CNTs filled with a single line of water molecules. As before with H_3_O^+^,^15^ we observed an enhancement in the 1D diffusion coefficient of OH^–^. The comparison is only qualitative, as neither experimental data nor AIMD data are available in pristine nanotubes. The 3AP model provides OH^–^ mobility similar to that obtained with a dissociative water model.^71^

Although the 3AP model has a solvation structure conducive to proton hopping, the C parameter required in order to obtain experimental proton hopping rates and diffusion coefficient is quite high (35 kcal/mol), significantly higher than the *C* of 20–23.5 kcal/mol required for H_3_O^+^.^15^ This is likely caused by the high distance sensitivity of the strong water–hydroxide interactions. The optimal OO distance of water–hydroxide H bonds is ∼2.4 Å, whereas the same distance in water–water H bonds is ∼2.8 Å. Thus, instantaneous interconversion of OH and HOH causes a large increase in energy due to these distance mismatches, as observed previously for H_3_O^+^.^78^ Posthop energy minimization is then required to remove the excess energy added to the system upon an accepted proton hop. Polarizable water models might be able to reduce the proton hopping energies but are computationally expensive and have not been exhaustively tested against biomolecular force fields. The models proposed here pave the way to studies of possible OH^–^-mediated proton hopping in biomolecular systems, such as the Hv1 proton channel^79^ and bacteriorhodopsin.^80^

## Supporting Information Available

Details of the nonbonded parameter optimization; tables including updates to the modified Lee and Meuwly parameter set traced over 8 iterations of the SD algorithm and different placements of the charge and nonbonded parameters for 4AP models; and supplementary figures including O_H_–O_W_ RDF, statistical uncertainty in the RDFs, changes in the O_H_–O_W_ RDFs, χ^2^ values, 5CN solvation structure, RDF comparisons, mean square distance as a function of time, and triplicates of the distance vs time obtained from a 100 ps trajectory (PDF)
