# Supplementary material for: Classical Models of Hydroxide for Proton Hopping Simulations
Source: J Phys Chem B. 2024 Dec 3;128(49):12161–70. doi: 10.1021/acs.jpcb.4c05499 (PMC11647885; doi:10.1021/acs.jpcb.4c05499)
Supplement: Supplementary file 1 — jp4c05499_si_001.pdf [file jp4c05499_si_001.pdf]

Supporting Information

Classical Models of Hydroxide for Proton Hopping Simulations

Ankita Dutta[1,2] & Themis Lazaridis[1,2,3]*

1. Department of Chemistry and Biochemistry,
City College of New York/CUNY,
160 Convent Ave, New York, NY 10031, USA

2. Graduate Program in Biochemistry
The Graduate Center, City University of New York,
365 Fifth Ave., New York, NY 10016, USA

3. Graduate Programs in Chemistry and Physics
The Graduate Center, City University of New York,
365 Fifth Ave., New York, NY 10016, USA

* Tel. (212) 650-8364   Email: tlazaridis@ccny.cuny.edu

Nonbonded parameter Optimization

Prior to applying the SD algorithm, the derivative of an observable with respect to nonbonded parameters of interest ($\frac{dA}{d\lambda}$) is obtained based on previous work [1]

$$\frac{\mathrm{d}}{\mathrm{d}\lambda}\langle A\rangle_\lambda = \frac{1}{Q(\lambda)}\int A(\mathbf{r}, V)\exp(-\beta(E(\mathbf{r}, V;\lambda) + PV))\left(-\beta\frac{\mathrm{d}E(\mathbf{r}, V)}{\mathrm{d}\lambda}\right)\mathrm{d}\mathbf{r}\,\mathrm{d}V - \frac{1}{Q(\lambda)^2}\frac{\mathrm{d}Q}{\mathrm{d}\lambda}\int A(\mathbf{r}, V)\exp(-\beta(E(\mathbf{r}, V;\lambda) + PV))\,\mathrm{d}\mathbf{r}\,\mathrm{d}V$$

$$= -\beta\left(\left\langle A\frac{\mathrm{d}E}{\mathrm{d}\lambda}\right\rangle_\lambda - \langle A\rangle_\lambda\left\langle\frac{\mathrm{d}E}{\mathrm{d}\lambda}\right\rangle_\lambda\right) \quad \text{.......(S1)}$$

where $\beta = \frac{1}{k_B T}$, A is the observable and the dE/dλ is the derivative of the potential energy with respect to the given parameter. The <> brackets signify ensemble averages. Here the A observables are the coordination number (CN) and the height of the first peak of the O_H-O_W RDF. Instantaneous CN was calculated by obtaining the number of all O_W (water oxygens) within a cutoff distance of 3.1 Å from the O_H atom (OH^- oxygen). The instantaneous peak height

was obtained by calculating the number of O_W within an annulus between 2.35 and 2.75 Å from the O_H atom and dividing by the volume of the annulus.

Here, λ includes the well depth and the radius of hydroxide oxygen and hydrogen ($\mathcal{E}_o$, $\mathcal{E}_H$, R_{minO}, R_{minH}) in the Lennard-Jones potential (equation S2).

$$E_{LJ} = \sum_{nb} \varepsilon_{ij}\left[\left(\frac{r_{min_{ij}}}{r_{ij}}\right)^{12} - 2\left(\frac{r_{min_{ij}}}{r_{ij}}\right)^{6}\right] \qquad \text{.......(S2)}$$

Standard CHARMM (Lorentz-Berthelot) combination rules are applied

$$\varepsilon_{ij} = \sqrt{\varepsilon_i * \varepsilon_j} \qquad \text{.......(S3)}$$

$$r_{min_{ij}} = \frac{r_{min_i} + r_{min_j}}{2} \qquad \text{.......(S4)}$$

Equation S2 can be rewritten as follows:

$$E_{LJ} = \sum_{nb} \sqrt{\varepsilon_i * \varepsilon_j}\left[\left(\frac{r_{min_i} + r_{min_j}}{2r_{ij}}\right)^{12} - 2\left(\frac{r_{min_i} + r_{min_j}}{2r_{ij}}\right)^{6}\right] \qquad \text{.......(S5)}$$

The derivative of the potential energy with respect to parameter ε_i is

$$\frac{dE_{LJ}}{d\varepsilon_i} = \sum_{nb} \frac{1}{2\sqrt{\varepsilon_i}}\sqrt{\varepsilon_j}\left[\left(\frac{r_{min_i} + r_{min_j}}{2r_{ij}}\right)^{12} - 2\left(\frac{r_{min_i} + r_{min_j}}{2r_{ij}}\right)^{6}\right] \qquad \text{.......(S6)}$$

and that with respect to parameter r_{min_i} or r_{min_j} is

$$\frac{dE}{dr_{min_{ij}}} = 3\sqrt{\varepsilon_i * \varepsilon_j} * \left(\sum_{nb} \frac{(r_{min_i} + r_{min_j})^{11}}{1024 r_{ij}{}^{12}} - \sum_{nb} \frac{(r_{min_i} + r_{min_j})^{5}}{16 r_{ij}{}^{6}}\right) \qquad \text{.......(S7)}$$

The subscripts 'i' and 'j' associated with each LJ parameter refer to the individual atoms of interest in a given atom pair. Here, atom pairs of interest correspond to the oxygen and hydrogen atoms of a standard TIP3P model and those of a classical 2p OH^- model. The r_{ij} refers to the

distances between the atom pair of interest and is determined using the atomic coordinates obtained from the MD trajectories.

Table S1. Updates to the modified Lee & Meuwly parameter set (I1) traced over 8 iterations of the SD algorithm. I6 corresponds to the local minimum of χ^2 observed in Figure S4a. Units are kcal/mol for ε and Å for R_{min}.

	**I1**	**I2**	**I3**	**I4**	**I5**	**I6**	**I7**	**I8**
ε_o	0.0700	0.0701	0.0700	0.0700	0.0699	0.0699	0.0698	0.0698
ε_H	0.0410	0.0410	0.0410	0.0410	0.0410	0.0410	0.0410	0.0410
**$Rmin_O$**	1.6000	1.6059	1.6031	1.6000	1.5961	1.5990	1.5976	1.5953
**$Rmin_H$**	1.9500	1.9781	1.9887	2.0068	2.0816	2.1051	2.1238	2.1238
**CN**	5.0700	5.0466	5.0090	4.9166	5.1466	5.0449	5.0850	4.9830
**Peak**	8.5400	6.9954	7.1947	6.7125	5.7729	5.6516	5.6395	5.8300

Table S2: Different placements of the charge and nonbonded parameters for 4AP models.

	**Placement (1)**	**Placement (2)**	**Placement (3)**
**q_o (e)**	-	-	+0.2000
**q_H (e)**	+0.0742	+0.0742	+0.0742
**q_{AP} (e)**	-0.2685	-0.2685	-0.3185
**ε_o (kcal/mol)**	-	-0.0240	-0.0240
**ε_H (kcal/mol)**	-0.0460	-0.0460	-0.0460
**ε_{AP} (kcal/mol)**	-0.0060	-	-
**$Rmin_o$ (Å)**	-	1.9825	1.9825
**$Rmin_H$ (Å)**	0.2245	0.2245	0.2245
**$Rmin_{AP}$ (Å)**	1.2825	-	-

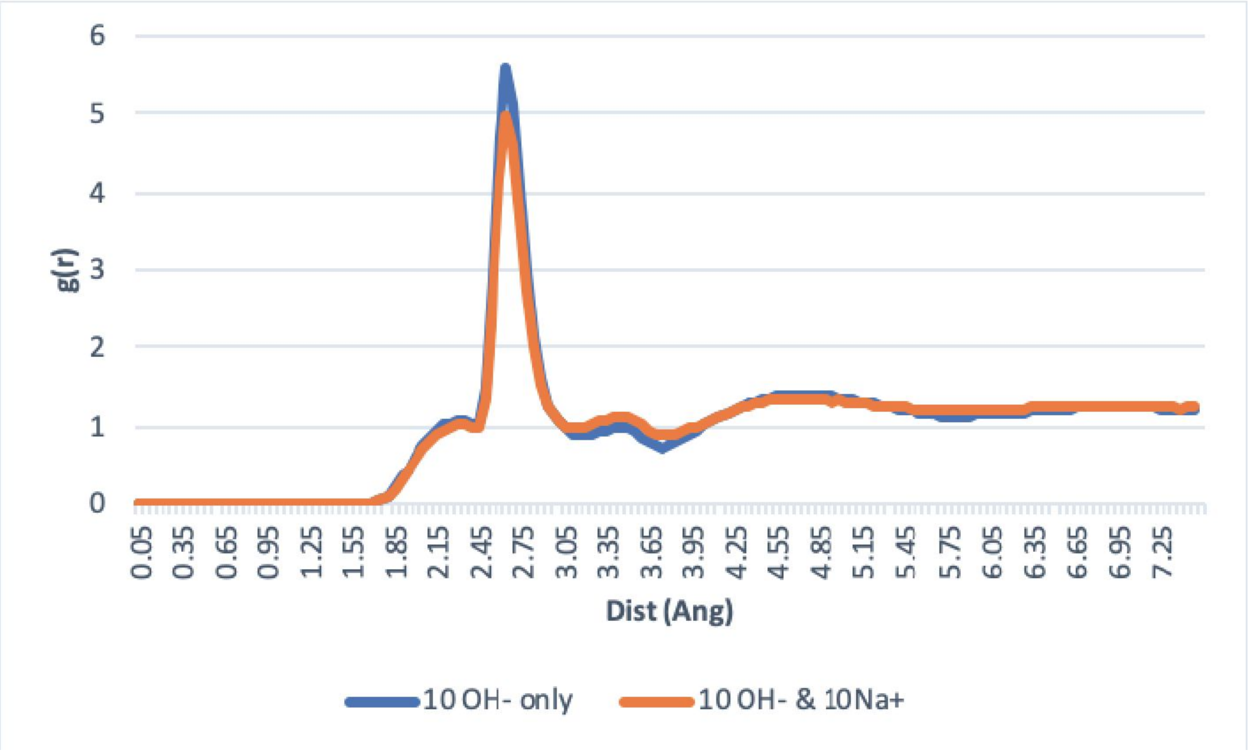

Figure S1. O_H-O_W radial distribution function in a system with ten OH^- with (orange) and without (blue) ten Na^+ counterions.

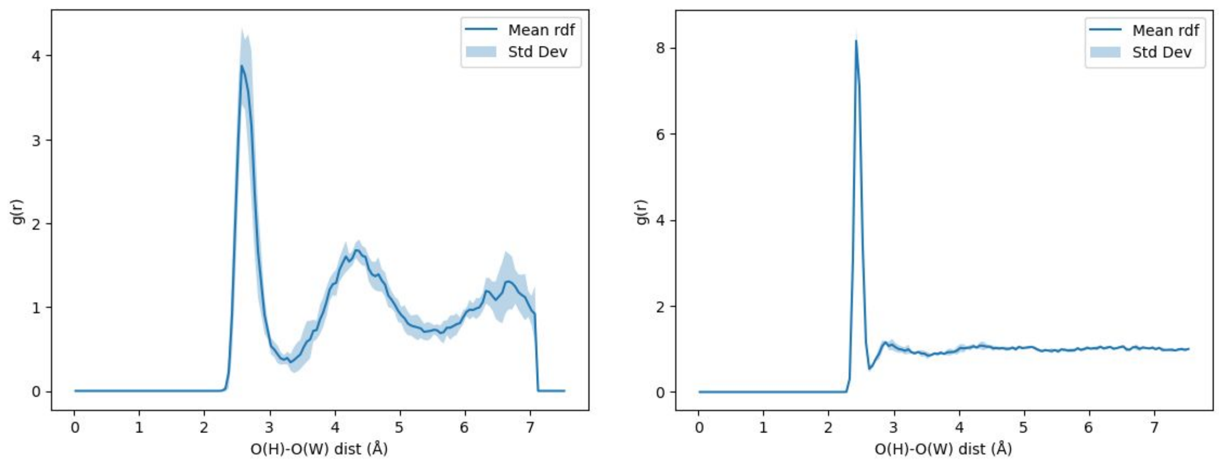

Figure S2. Statistical uncertainty in the RDFs calculated by splitting each trajectory in four blocks: a) AIMD trajectory, b) classical trajectory.

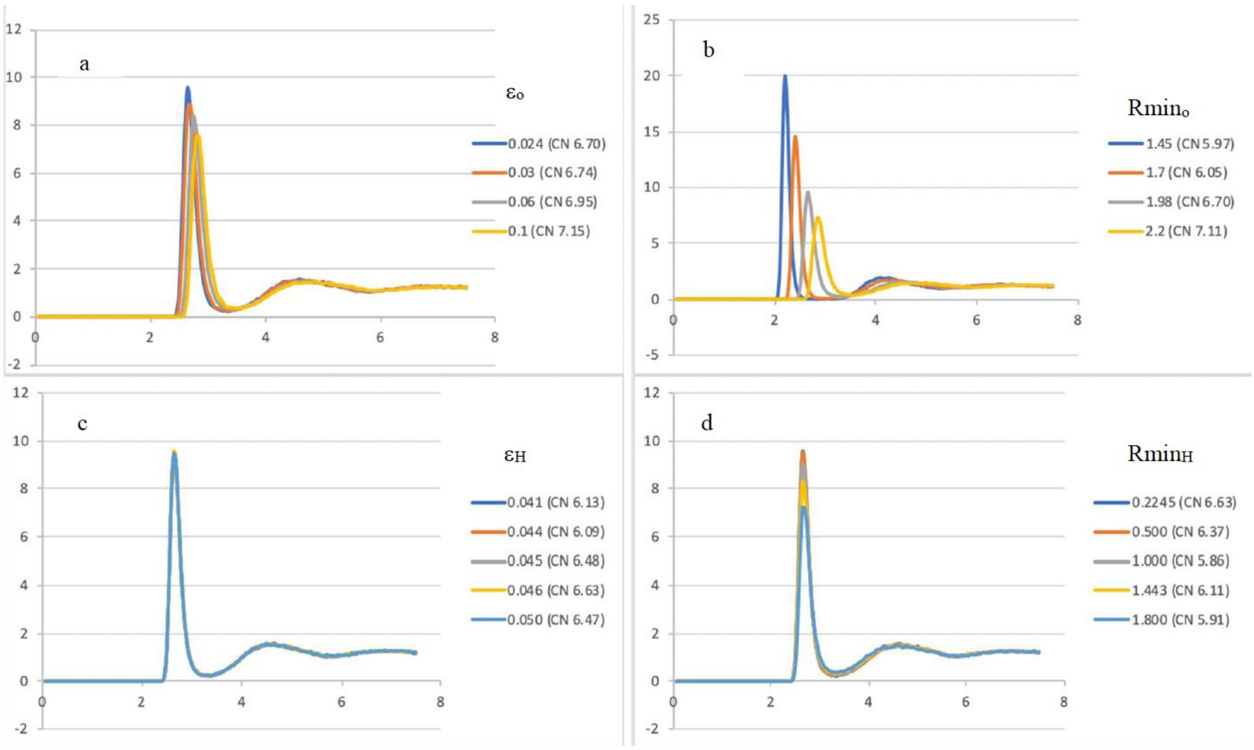

Figure S3. Changes in the O_H-O_w RDFs observed when a given oxygen or hydrogen LJ parameter is modified independent of other force field parameters.

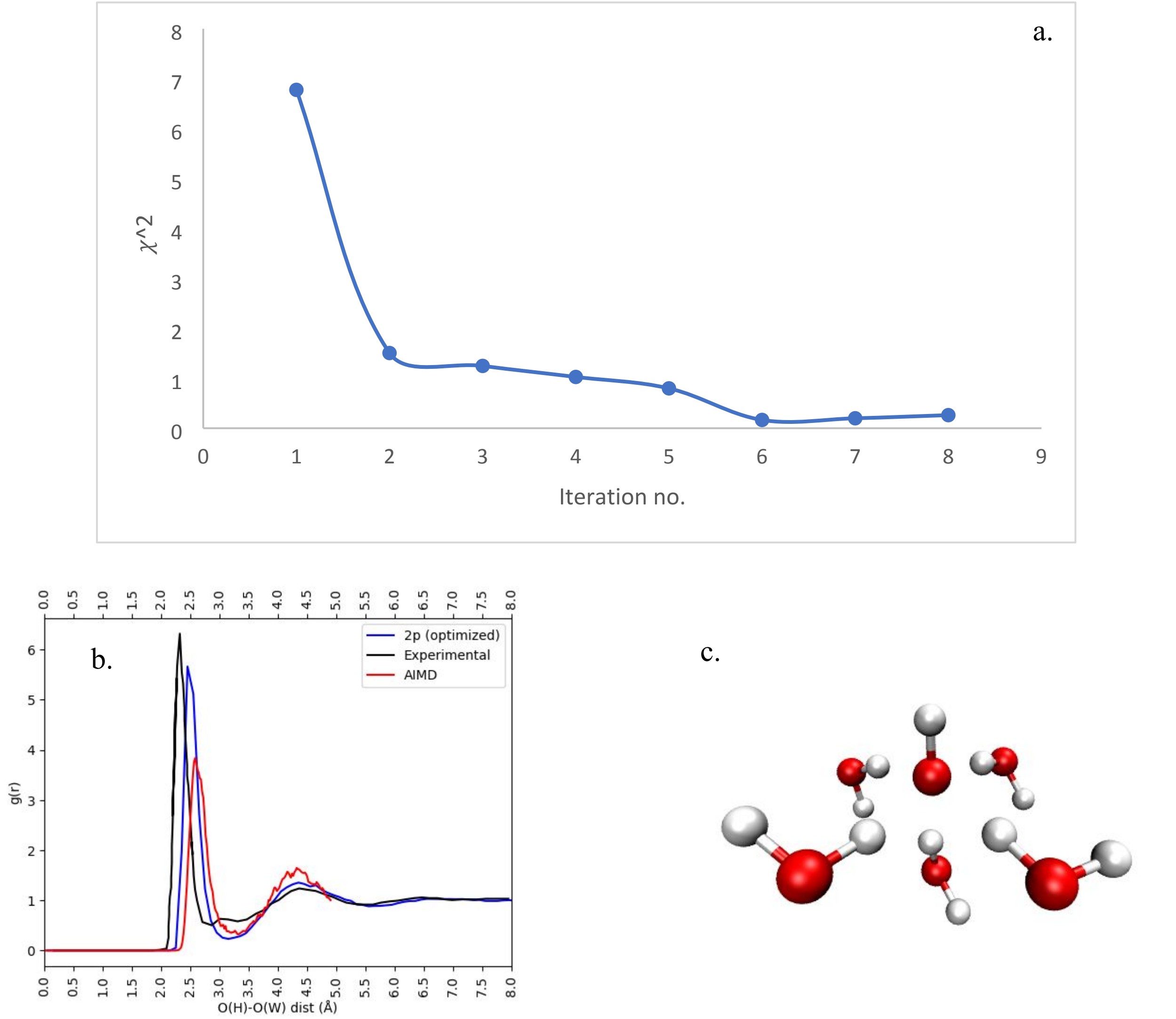

Figure S4. a) χ^2 values obtained over several iterations of the SD algorithm starting with the modified Lee-Meuwly parameter set. b) Comparison of O_H-O_W RDF obtained at the χ^2 minimum with those obtained using experiment (Figure 6 of Ref [2]) and the analysis of the AIMD trajectory. c) 5CN solvation structure observed at the χ^2 minimum. 1 water molecule is present in a nonplanar arrangement with respect to the rest of the water molecules in the first hydration shell.

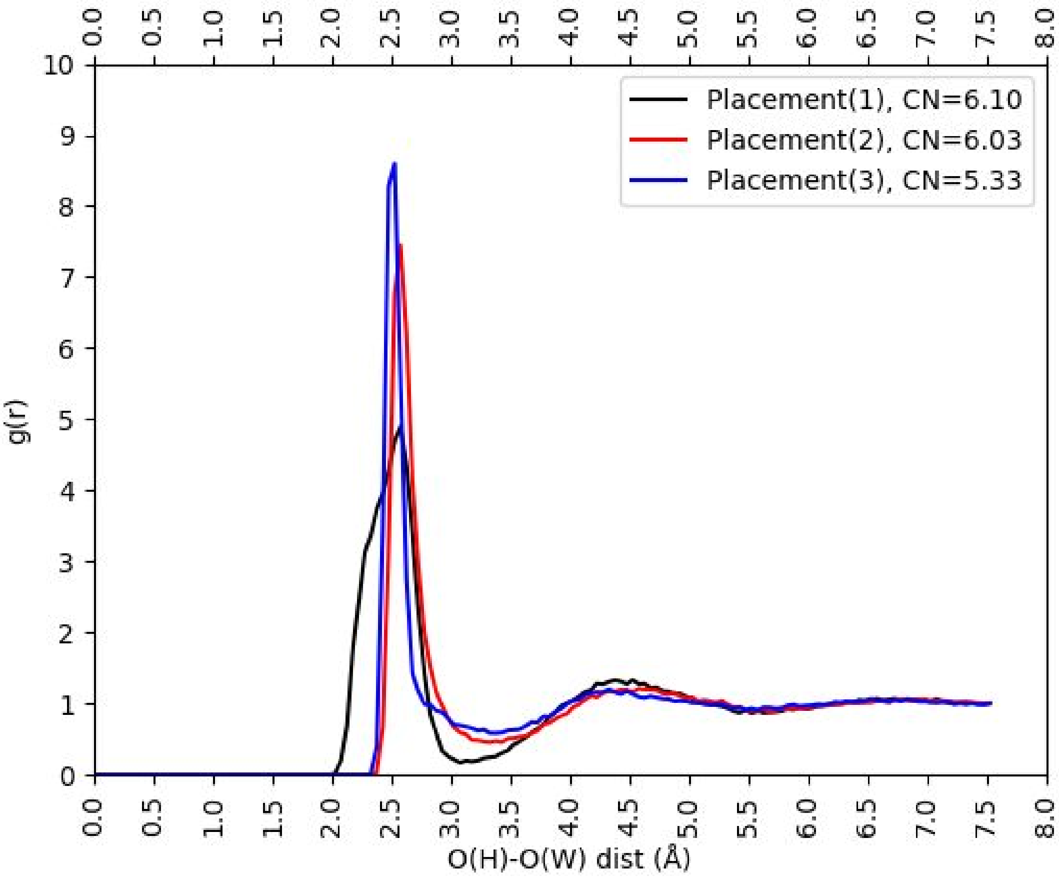

Figure S5. Changes in the O_H-O_W RDFs observed with different placements of nonbonded parameters of the oxygen atom in a 4AP model with overall charge of -1.0

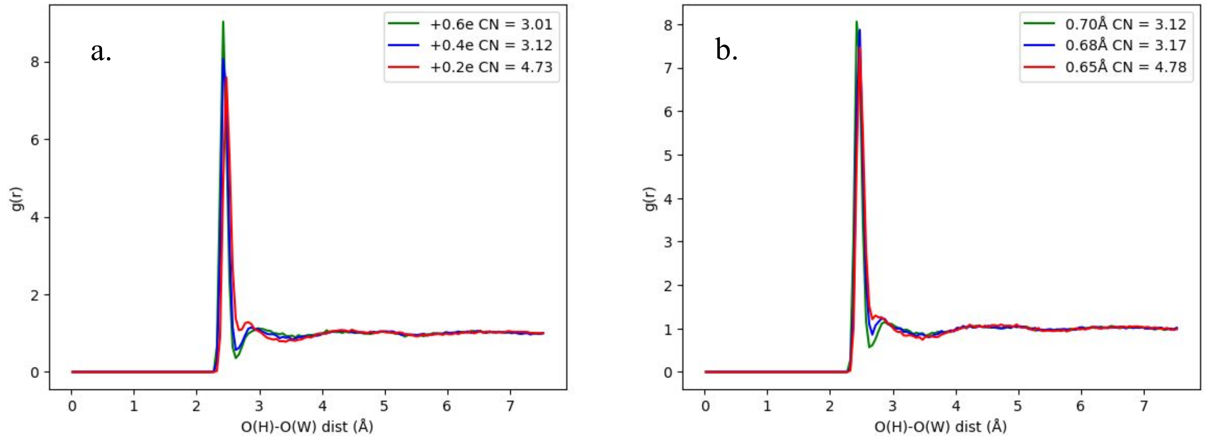

Figure S6. Changes in the O_H-O_W RDFs observed using a 3AP model with overall charge of -0.9 with a) increasing magnitude of positive charge assigned to the oxygen atom and b) increasing O-AP bond length.

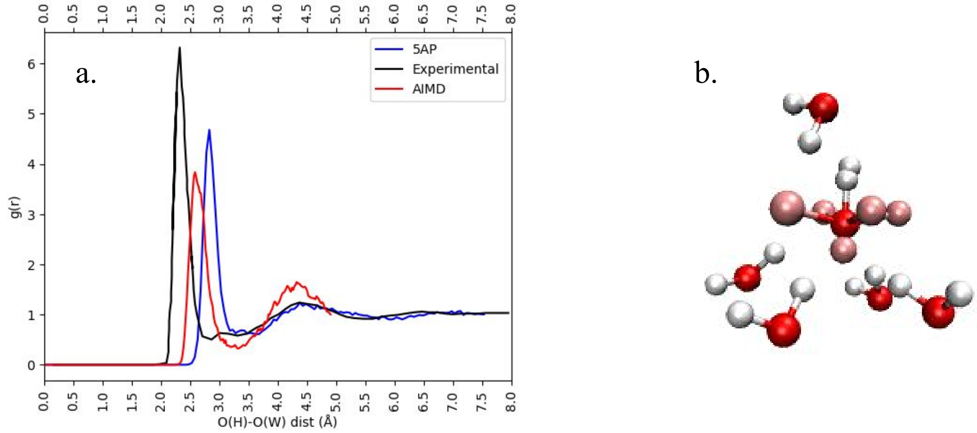

Figure S7. a) Comparison of O_H-O_W RDF obtained with the 5AP model with an overall charge of -1.0e, to those obtained using experiment (Figure 6 of Ref [2]) and AIMD. b) Representative solvation structure around the 5AP model.

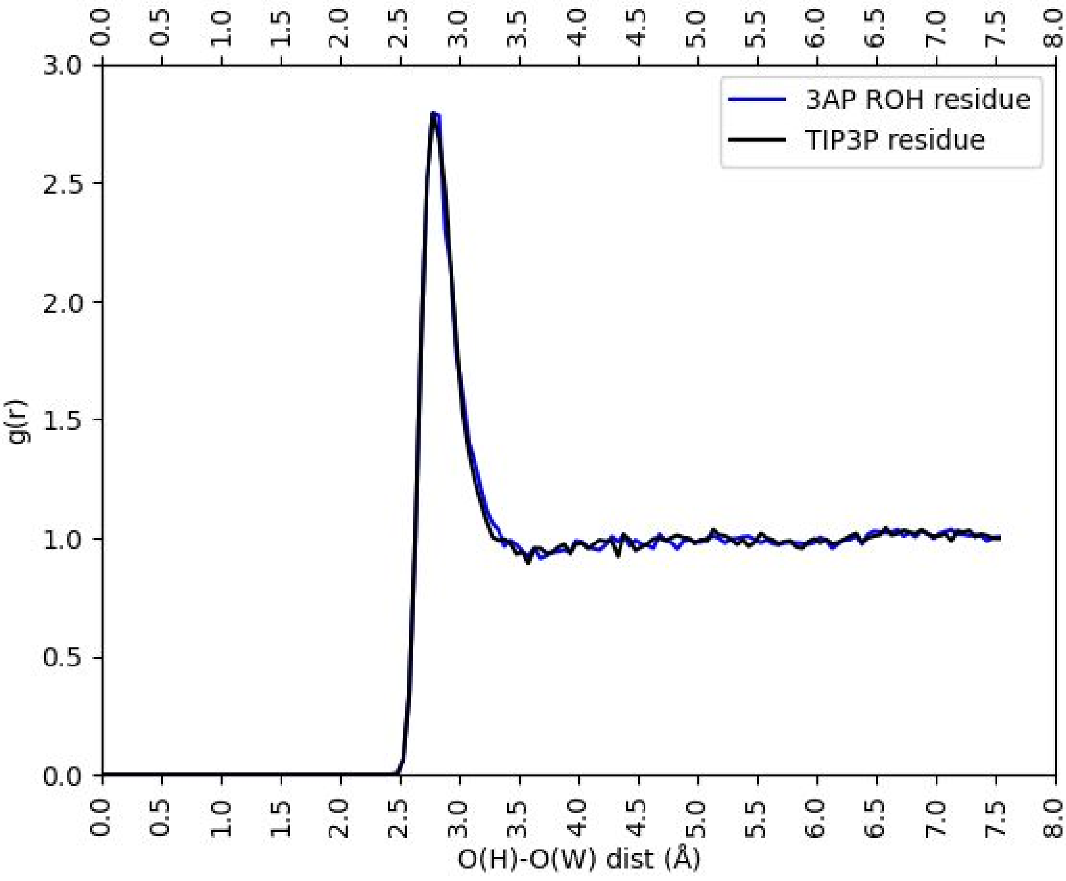

Figure S8. RDF comparison of non-titratable TIP3P waters and protonated HOH residues

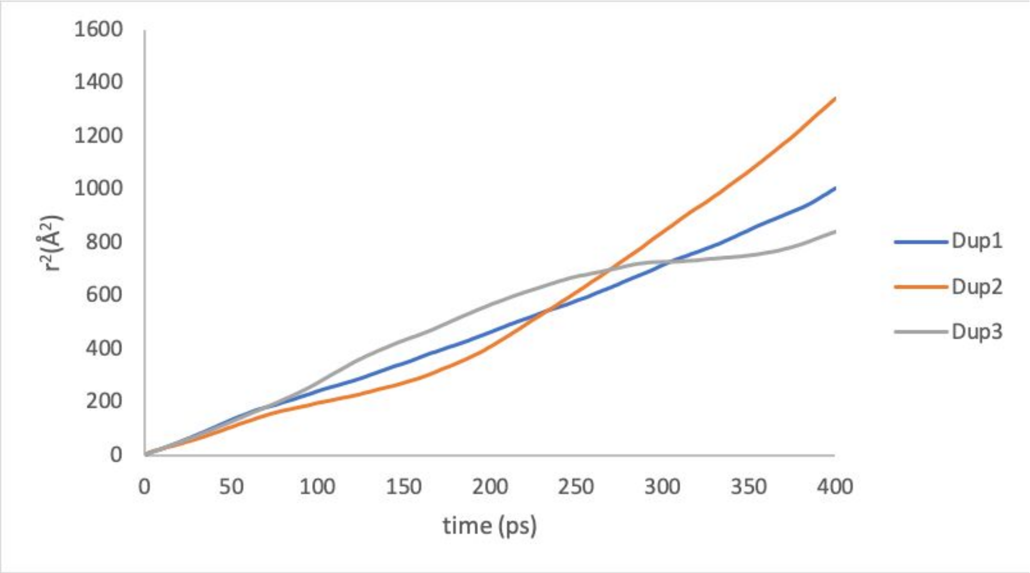

Figure S9. Mean square distance as a function of time for OH- diffusion with threshold parameter C=35 kcal/mol. The slope is calculated from 0 to 40 ps.

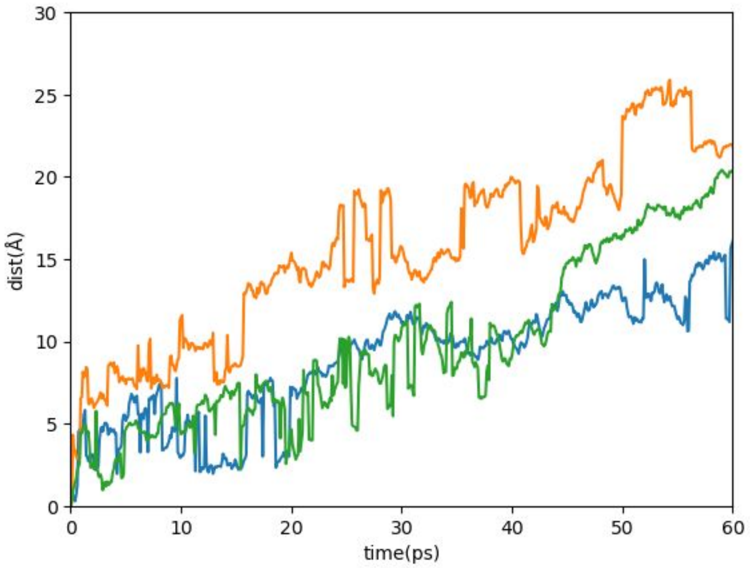

Figure S10. Triplicates of the distance vs time obtained from a 100 ps trajectory (the first 60 ps is plotted here) with external potential equivalent to 1.1V applied.

References

[1] Wang, L. P., Head-Gordon, T., Ponder, J. W., Ren, P., Chodera, J. D., Eastman, P. K., ... &

Pande, V. S. (2013). Systematic improvement of a classical molecular model of water. The Journal of Physical Chemistry B, 117(34), 9956-9972

[2] McLain, S. E., Imberti, S., Soper, A. K., Botti, A., Bruni, F., & Ricci, M. A. (2006). Structure of 2 M NaOH in aqueous solution from neutron diffraction and empirical potential structure refinement. Physical Review B, 74(9), 094201
